# Pituitary Abscess Syndrome in Ruminants: Nine Cases

**DOI:** 10.3390/ani15182692

**Published:** 2025-09-15

**Authors:** Gabriele Maroneze, Liz de Albuquerque Cerqueira, José Renato Junqueira Borges, Márcio Botelho de Castro, Antonio Carlos Lopes Câmara

**Affiliations:** 1 Large Animal Veterinary Teaching Hospital, College of Agronomy and Veterinary Medicine, Universidade de Brasília, Brasilia 70636-200, DF, Brazil; gabimaronezi@gmail.com (G.M.); jrborges@unb.br (J.R.J.B.); 2 Veterinary Pathology and Forensic Laboratory, College of Agronomy and Veterinary Medicine, Universidade de Brasília, Brasilia 70910-900, DF, Brazil; lizcerqueira@hotmail.com (L.d.A.C.); mbcastro@unb.br (M.B.d.C.)

**Keywords:** empyema basilar, livestock, pituitary gland, suppurative intracranial processes

## Abstract

Pituitary abscess syndrome (PAS) is a rare neurological disorder, typically associated with progressive dysfunction of the cerebrum and brainstem. Reporting PAS in ruminants is essential to broaden the global veterinary understanding of this condition. The present study describes the epidemiological, clinical, laboratory, microbiological, and pathological findings of nine ruminant cases diagnosed with PAS. Neurological signs were the most prominent clinical manifestations and included altered mentation, tongue hypotonia, nystagmus, blindness, ear ptosis, circling, facial hypoalgesia, head pressing, and proprioceptive deficits. Hematological alterations in some animals comprised leukocytosis by neutrophilia, a degenerative left shift, and hyperfibrinogenemia. Serum biochemical abnormalities were inconsistent and varied among cases. Cerebrospinal fluid (CSF) analysis revealed marked variability, ranging from normal parameters to mild or marked pleocytosis, often accompanied by hyperproteinorrachia. Microbiological cultures from CSF samples or abscess material yielded *Trueperella pyogenes*, *Streptococcus* spp., and *Corynebacterium* spp. Gross pathological findings primarily included pituitary hyperemia, abscess formation, or diffuse suppurative inflammation characterized by a creamy yellow to greenish exudate. Histopathological examination revealed severe multifocal suppurative inflammation composed predominantly of neutrophils, occasional histiocytes, abundant bacteria, areas of necrosis, and encapsulated abscesses. This retrospective study provides novel insights into the clinical, laboratory, and pathological characteristics of PAS in ruminants under field conditions, thereby contributing to improved recognition and diagnostic understanding of this uncommon disease.

## Simple Summary

This study provides essential knowledge of pituitary abscess syndrome (PAS), thereby contributing to the understanding of this uncommon neurologic condition in ruminants. In a 20-year survey, nine ruminants presenting mainly neurological signs were diagnosed with PAS. Despite intensive care, death or euthanasia was the outcome in all animals. A definitive diagnosis of PAS has been reached through a combination of epidemiological, clinical, laboratory, and pathological features.

## 1. Introduction

Central nervous system (CNS) diseases in ruminants represent a significant diagnostic challenge for veterinarians and are of global relevance [1]. Infections in the CNS are among the most lethal diseases and generally have a substantial economic impact on livestock production worldwide [2]. In adult ungulates, three uncommon suppurative intracranial processes are identified, including brain abscess, basilar empyema (pituitary abscess), and suppurative meningitis [3].

Pituitary abscess syndrome (PAS), also known as basilar empyema or cavernous sinus syndrome, is an uncommon neurologic disease typically characterized by progressive signs of cerebral and brainstem dysfunction [2]. Cases have been reported in cattle [4,5,6,7,8,9,10,11], goats [4,12,13], sheep [4,10,14,15], horses [16,17], and humans [18,19]. In this context, reporting PAS in ruminants with uncommon neurological presentations is crucial to expanding the global veterinary knowledge base. The present study aimed to describe the epidemiological, clinical, laboratory, microbiological, and pathological characteristics of nine cases of PAS in ruminants.

## 2. Materials and Methods

A 20-year retrospective survey (January 2005 to December 2024) was conducted using the medical records of ruminants referred to the Large Animal Veterinary Teaching Hospital of the Universidade de Brasília, located in Brasília, Federal District, Midwestern Brazil. Cases were included based on a conclusive diagnosis of PAS, established through a combination of epidemiological, clinical, laboratory, and pathological findings.

Epidemiological data included species (cattle, goats, or sheep), breed, and age. Clinical information, such as disease progression, physical examination results, and treatments, was extracted from the medical records. As part of the standard physical examination, a complete neurological assessment was performed [1]. Blood samples were collected via jugular venipuncture for hematological and serum biochemical analyses. Cerebrospinal fluid (CSF) was obtained from the atlanto-occipital space and submitted for cytological and biochemical evaluation [20,21,22].

Animals that died spontaneously or were euthanized for welfare reasons underwent necropsy. Tissues were fixed in 10% neutral buffered formalin, routinely processed, paraffin-embedded, sectioned at 5 μm, and stained with hematoxylin and eosin (H&E) for histopathological examination under light microscopy.

In selected cases, CSF aliquots or abscess swabs were collected aseptically and inoculated onto 8% sheep blood agar (Sigma-Aldrich, Darmstadt, Germany) and MacConkey agar (Neogen Corporation, São Paulo, Brazil), followed by aerobic incubation at 37 °C for 24–72 h. Simultaneously, samples were subjected to microaerophilic (5% CO_2_) and anaerobic culture on sheep blood agar and incubated at 37 °C for 120 h. Bacterial isolates were identified using conventional bacteriological methods, including assessment of colony morphology, pigment production, Gram staining, growth at 44 °C, and a panel of biochemical tests (oxidase, catalase, nitrate reduction, indole, methyl red, Voges–Proskauer, citrate utilization, and glucose fermentation), as described by Quinn et al. [23].

## 3. Results

During the 20-year study period, a total of 3546 ruminants (1626 cattle, 1435 sheep, and 485 goats) were admitted for hospital care. Among these, six cattle, two sheep, and one goat (totaling nine animals: 0.25%) were conclusively diagnosed with PAS. The frequency of PAS was 0.37% in cattle, 0.13% in sheep, and 0.20% in goats during routine hospital admissions. Epidemiological and clinical data are summarized in Table 1.

**Figure 1:**
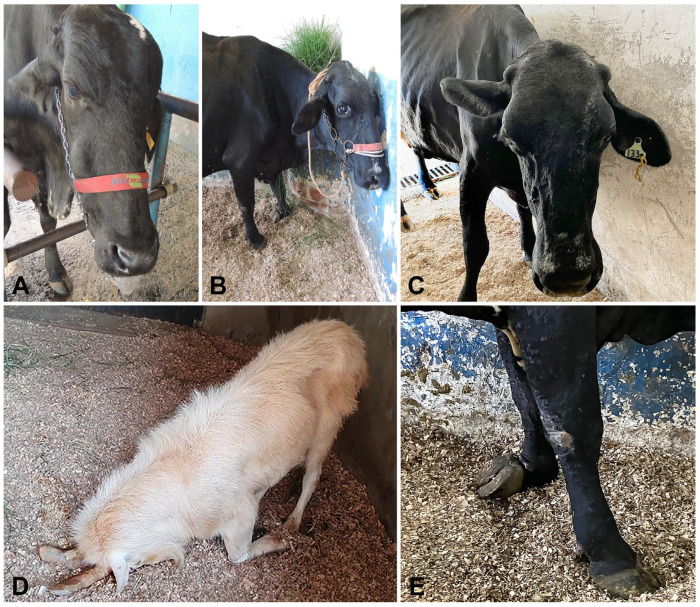
Clinical signs in ruminants with pituitary abscess syndrome. (**A**) Cow (Case 6). Hypotonia with the extrusion of the tongue. (**B**) Cow (Case 8). Blindness and accidental collision with the wall. (**C**) Cow (Case 8). Ptosis of the left ear. (**D**) Goat (Case 9). Head pressing against the ground. (**E**) Cow (Case 8). Proprioceptive deficit affecting the left front limb.

The most prominent clinical manifestations were neurological signs, including altered mentation, tongue hypotonia (Figure 1A), nystagmus, blindness (Figure 1B), ear ptosis (Figure 1C), circling, facial hypoalgesia, dropped jaw, head pressing (Figure 1D), proprioceptive deficits (Figure 1E), and recumbency accompanied by pedaling movements. Additional clinical findings in some ruminants included dehydration (assessed by skin turgor), fever, sialorrhea, purulent nasal discharge, and pulmonary crackles.

Laboratory data, including hematological and biochemical findings, are summarized in Table 2. Blood samples were unavailable for two cattle (Cases 3 and 4) that died on the farm prior to hospital admission. The laboratory results varied considerably among cases. Notable hematological alterations included marked leukocytosis with neutrophilia (Cases 2 and 9), inversion of the neutrophil/lymphocyte ratio (Case 7), eosinophilia (Case 6), lymphopenia, and a degenerative left shift characterized by immature neutrophils outnumbering segmented forms (Case 1) [24]. All affected ruminants exhibited hyperfibrinogenemia [24]. The most frequent biochemical abnormalities were hyperproteinemia (*n* = 2), hyperglobulinemia (*n* = 3), hypoalbuminemia (*n* = 3), increased activities of aspartate aminotransferase (AST; *n* = 2) and γ-glutamyl transferase (GGT; *n* = 2), and elevated serum urea (*n* = 2) and creatinine (*n* = 1) concentrations [25].

The CSF analysis results are presented in Table 3. Two cattle underwent CSF collection on the first day of hospitalization, revealing mild pleocytosis, neutrophilic in Case 1 and monocytic in Case 2, accompanied by hyperproteinorrachia [21,22]. In Case 1, microbiological culture of a CSF aliquot yielded *Trueperella pyogenes*. In one cow (Case 8) and one goat (Case 9), CSF was collected on the second day of hospitalization. The cow exhibited mild monocytic pleocytosis [22]. Following clinical deterioration, a repeat CSF analysis was performed in the cow prior to euthanasia on day 9, showing a cloudy appearance, yellow discoloration, marked neutrophilic pleocytosis, hyperproteinorrachia, and the presence of both intracellular and free bacteria. Microbiological culture of CSF from this cow yielded *Corynebacterium* sp. only during the second analysis. In contrast, CSF parameters from the goat (Case 9) remained within reference ranges in both evaluations [21].

**Figure 2:**
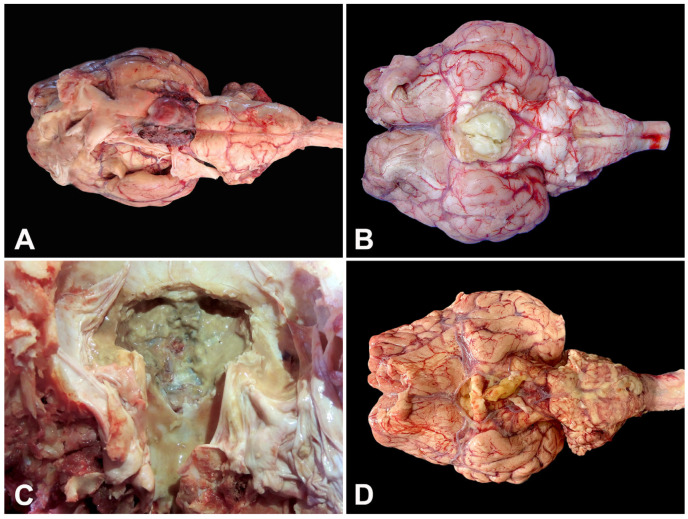
Gross findings in ruminants with pituitary abscess syndrome. (**A**) Goat (Case 9). Severe hyperemia surrounding the pituitary gland. (**B**) Calf (Case 1). Severe abscessation at the pineal gland. (**C**) Cow (Case 6). Suppurative greenish material within the *sella tursica* (pituitary fossa). (**D**) Cow (Case 8). Severe suppurative meningitis extending ventrally to the brainstem.

Despite intensive therapeutic interventions, including fluid therapy, broad-spectrum antibiotics, non-steroidal anti-inflammatory drugs, and corticosteroids in some high-value ruminants, the outcome was invariably death or euthanasia. The necropsy findings revealed prominent gross lesions, most notably hyperemia surrounding the pituitary gland (Figure 2A). Frequently, well-demarcated space-occupying lesions (abscesses) or diffuse suppurative inflammation were observed, characterized by creamy yellow (Figure 2B) or greenish exudate (Figure 2C) within the affected tissues.

**Figure 3:**
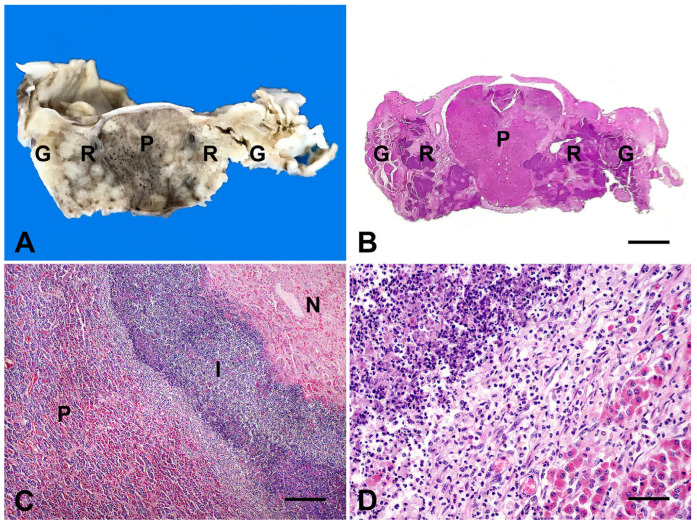
Gross and microscopic findings in ruminants with pituitary abscess syndrome. (**A**) Sheep (Case 5). Whitish spots representing multifocal suppurative inflammation affecting glandular (P: pituitary gland), vascular (R: *rete mirabile carotidea*), and nervous tissues (G: trigeminal ganglia). (**B**) Sheep (Case 5). Severe suppurative inflammation and multifocal abscessation affecting the pituitary gland (P), *rete mirabile carotidea* (R), and trigeminal ganglia (G). (H&E, bar = 1000 µm). (**C**) Cow (Case 3). Extensive area of necrosis (N), suppurative inflammation (I) severely affecting the pituitary tissues (P). (H&E, bar = 250 µm). (**D**) Cow (Case 8). Neutrophils, necrotic tissues, and fibrosis are effacing the pituitary tissues. (H&E, bar = 50 µm.)

In several cases, severe suppurative meningitis extended ventrally to the brainstem (Figure 2D) or dorsally into abscesses compressing the hypothalamus and thalamus. The *rete mirabile carotidea* was commonly involved, allowing multifocal extension of the inflammatory process to the pituitary gland and trigeminal ganglion (Figure 3A).

The most pronounced microscopic lesions in ruminants affected by PAS involved the pituitary gland, the *rete mirabile carotidea*, and, in some cases, the trigeminal ganglia (Figure 3B). These structures were extensively infiltrated and disrupted by severe, multifocal inflammation (Figure 3C), predominantly composed of neutrophils with occasional histiocytes (Figure 3D), accompanied by abundant bacteria, areas of necrosis, and encapsulated abscesses. Surrounding neural tissues frequently exhibited fibrinosuppurative meningitis, neuronal degeneration, gliosis, mononuclear perivascular cuffing, and edema.

Additionally, swabs collected aseptically from pituitary abscesses in two cattle (Cases 3 and 8) were submitted for microbiological culture, resulting in the isolation of pure cultures of *Streptococcus* sp. and *Corynebacterium* sp., respectively.

## 4. Discussion

Pituitary abscess syndrome (PAS) is considered an uncommon suppurative intracranial process affecting ruminants, which is usually fatal [3,5,6,7,26]. This feature is confirmed by the low incidence in our hospital routine (0.25%—9 of 3546 ruminants), reiterating the rarity of PAS in ruminant clinics [2,3,26]. Historically, PAS has been described in outbreaks or sporadic cases in calves aged 3 to 12 months, as a consequence of traumatic rhinitis produced by the use of weaner nose rings [5,7,10,26], and metal nose rings in adult cattle [2,10,26]. The condition is even rarer in small ruminants, with only a few sporadic reports [12,14,15] or retrospective studies [4,10,13] worldwide.

Although the precise pathogenesis of PAS remains unclear [2,3,6,9,26], it is generally postulated that bacterial dissemination from distant infection foci occurs via the cavernous sinus that receives venous return not only from the CNS but also from vessels draining soft tissues of the head, such as the face, orbit, nasal cavity, and maxilla. Because its venous channels lack valves, blood can circulate in both directions, enabling flow between these peripheral regions and the cavernous and intercavernous sinuses and their branches [27]. This particular venous architecture is considered a risk factor for the deposition of septic emboli and subsequent abscess development, which may compromise the pituitary, its vascular network, and neighboring neural structures [4,5,7]. This mechanism may explain several cases in the present study, in which five ruminants presented concurrent infected lesions in the head (nasal septum, oral mucosa, mandible, or horns), while two others had distant infection foci (bronchopneumonia or mastitis).

Clinical signs reported in the ruminants herein were almost exclusively confined to the nervous system, reflecting cerebral and brainstem involvement. Most manifestations were attributable to pressure exerted by abscesses on surrounding structures, resulting in unilateral or bilateral cranial nerve (CN) deficits or hemiplegia, particularly affecting CN II (optic), CN III (oculomotor), CN V (trigeminal), CN VI (abducent), CN VIII (vestibulocochlear), and CN XII (hypoglossal) [1,5,7,8,9]. Variability in clinical presentation is explained by abscess location, which may initially involve the vascular complex and/or pituitary gland and subsequently extend to the meninges, cerebellum, and brain parenchyma [3,7]. Identification of chronic infection sites within the head or at distant locations in ruminants exhibiting consistent neurological signs may increase the likelihood of a presumptive PAS diagnosis [1,2,26].

Hematological alterations included leukocytosis with neutrophilia and a degenerative left shift, along with hyperfibrinogenemia, are consistent with acute bacterial inflammation [24]. Biochemical abnormalities (hyperproteinemia, hyperglobulinemia, hypoalbuminemia, elevated AST and GGT) were variable and may be influenced by anorexia and recumbency [2,3,25]. Recently, evaluation of the serum levels of anterior pituitary hormones (thyroid stimulating hormone, follicle stimulating hormone, luteinizing hormone, and prolactin) and detection of electrolyte imbalances (hyponatremia, hypochloremia, hypokalemia) have been proposed as useful adjuncts to enhance the presumptive diagnosis of PAS in ruminants with neurological signs [11,14].

CSF analysis is an essential component of the diagnostic evaluation of ruminants presenting with CNS signs [3,21,22,28]. In the present study, the CSF results were available in only four clinical records. Among these, mild pleocytosis and hyperproteinorrachia were observed in three and two cattle, respectively. Notably, one cow subjected to serial CSF sampling demonstrated a marked increase in leukocyte count and protein concentration, with visible bacteria detected seven days later. This sample yielded a positive microbiological culture for *Corynebacterium* sp., despite ongoing antimicrobial therapy. In contrast, the Saanen buck (Case 9) exhibited no changes in the CSF parameters in both evaluations. These findings highlight the considerable variability reported in CSF alterations in ruminants with PAS [2,3,22,28], which may range from normal, indicating a non-suppurative response, to overtly purulent with bacterial presence [3]. For instance, a recent case in a bull showed markedly increased mixed pleocytosis (2437 cells/μL) and hyperproteinorrachia (3.1 g/L) [9]. Furthermore, no consistent differences in CSF parameters were observed among cattle with pituitary abscesses, vertebral body abscesses, or spinal epidural abscesses, with protein concentrations and cell counts varying from normal to markedly elevated [22].

Importantly, CSF analysis remains valuable for excluding other CNS infections that may present with similar neurological signs, particularly viral infections [29], for providing material for additional diagnostic assays (e.g., microbiological culture or viral isolation), and for monitoring treatment response [3,22,28]. By contrast, recent evidence suggests that the type of pleocytosis may not reliably differentiate disease forms in small ruminants [30].

Microbiological assays performed on CSF aliquots or abscess swabs in three cases yielded *T. pyogenes*, *Streptococcus* sp., and *Corynebacterium* sp. *T. pyogenes* is the most frequently reported pathogen in PAS of cattle [4,5,6,7,8,10,11], and occasionally in sheep [4,10], and is considered the primary agent in chronic suppurative CNS lesions in cattle [2,3,26]. Other bacteria, including *Staphylococcus* sp., *Streptococcus* sp., *Fusobacterium necrophorum*, *Corynebacterium pseudotuberculosis*, and *Mycoplasma arginini*, have also been associated with PAS in ruminants [2,3,12,13,26].

Antemortem diagnosis of PAS remains challenging, and a presumptive diagnosis is generally based on epidemiological context and neurological examination. Recently, computed tomography (CT) of a bull with a pituitary gland abscess revealed a focal lesion in the thalamus. Combined with CSF analysis, this lesion was interpreted as an abscess in the ventral thalamus, immediately rostral and dorsal to the hypophysis [9]. Advanced imaging modalities, including CT and magnetic resonance imaging, can substantially assist in the diagnosis of PAS, particularly by delineating the size, number, and precise anatomical location of abscesses [3,9]. Wider accessibility of these imaging technologies in veterinary practice would enhance early detection and management of PAS cases globally.

Definitive diagnosis of PAS is most often achieved postmortem [2,3,26], as observed in all cases reported herein. Gross and microscopic pathological findings in the affected ruminants were characteristic of PAS and consistent with previous reports [4,5,6,7,8,10,11]. The space-occupying nature of these suppurative CNS lesions disrupts the normal architecture of the pituitary gland, *rete mirabile carotidea*, trigeminal ganglia, and surrounding neural tissues, providing a plausible explanation for the extensive neurological deficits observed in the present cases.

Treatment of livestock with CNS infections, such as meningoencephalitis, is effective primarily when initiated early in the disease course. Once neurological signs have persisted for several days, or if recumbency or paralysis has developed, therapeutic interventions are typically unrewarding [2,31]. Management of CNS abscesses is particularly challenging and is often not recommended [2,3,26]. Nonetheless, a recent report described successful antimicrobial therapy in a bull, consisting of gentamicin for three days followed by amoxicillin for 40 days [9].

Although the prognosis of PAS is generally poor [3], as observed in the present cases, it is influenced by multiple factors, including abscess size, wall thickness, the causative bacterial species, and the anatomical location of the lesion [9]. Other reports have documented favorable outcomes using a combination of penicillin and streptomycin, although specific treatment durations were not provided [5,7].

Given the anatomical arrangement of the arteriovenous complex, which predisposes the *rete mirabile carotidea* and pituitary gland to abscess formation, the most rational preventive measures include strict aseptic technique during nose ring insertion and prompt identification and treatment of bacterial infections in other body systems [2,26]. Additionally, producers should be aware of the potential risk of PAS associated with the use of weaner nose rings. If their use is unavoidable, these devices should be applied with meticulous hygienic care, and the portions contacting the nasal septum should be polished to minimize traumatic effects [5,7,10]. Such measures may contribute to reducing the incidence of pituitary abscess formation.

The main limitations of this study were the small sample size and the absence of standardized laboratory protocols, particularly for microbiological assays. Nonetheless, the findings reported herein provide valuable field-based data on PAS in ruminants. Because PAS can manifest with sudden and severe neurological signs, many cases may not reach veterinary attention, potentially leading to underreporting or misdiagnosis. Therefore, this retrospective study offers critical insights into the clinical presentation, laboratory findings, and pathological features of this uncommon neurological disorder in ruminants.

## 5. Conclusions

In the present study, definitive diagnosis of pituitary abscess syndrome (PAS) was achieved through an integrated evaluation of epidemiological, clinical, laboratory, and pathological findings, providing valuable information for veterinarians worldwide regarding this uncommon neurological disorder. Most ruminants affected by PAS in this series also exhibited concurrent infectious foci in other body systems. Our findings underscore the severe clinical and laboratory alterations associated with the disease, as well as the significant negative impact on animal welfare. Furthermore, therapeutic interventions were unsuccessful in all attempted cases, highlighting the inherent challenges of treatment and supporting the view that it is often unrewarding or inadvisable.

## Figures and Tables

**Table 1:** Epidemiological data and clinical aspects from 9 ruminants (6 cattle, 2 sheep, and 1 goat) with pituitary abscess syndrome.

Case	Species	Breed	Age	Month/Year	Clinical Evolution ^a^	Risk Factors	Clinical Signs	Treatment
1	Cattle	Tabapuã	10 months	1/2005	20 days	Traumatic rhinitis (weaner nose ring)	Depression, fever, sialorrhea, purulent nasal discharge, head pressing	Penicillin (40,000 UI/kg)
2	Cattle	Holstein	11 months	8/2005	10 days	Bronchopneumonia	Depression, dehydration, hypotonic tongue, nystagmus, miosis, pedaling movements, opisthotonus, sialorrhea, pulmonary crackles	None
3	Cattle	Nelore	4 years	9/2011	3 days	Gingival fistula	Blindness, recumbency	Fluids and thiamine (10 mg/kg)
4	Cattle	Girolando	3 years	10/2016	4 days	NAD	Blindness, circling, sialorrhea, recumbency	NAD
5	Sheep	Crossbred	4 years	4/2019	24 h	None	Dehydration, nystagmus, opisthotonus, recumbency, pedaling movements	None
6	Cattle	Girolando	3 years	5/2019	20 days	Mandibular abscess	Face hypoalgesia, dropped jaw, tongue protrusion, sialorrhea	Sulphadimethoxine plus trimethopim (15 mg/kg), flunixin meglumine (2.2 mg/kg)
7	Sheep	Crossbred	3 years	6/2021	3 days	None	Depression, purulent nasal discharge, opisthotonus, recumbency	Fluids and meloxican (0.6 mg/kg)
8	Cattle	Girolando	7 years	5/2023	2 days	Gingival ulcer/mastitis	Depression, hypermetria, hypotonic tongue, proprioceptive deficits, head pressing, left auricular and palpebral ptosis	Florfenicol (20 mg/kg), dexamethasone (0.1 mg/kg)
9	Goat	Saanen	8 years	9/2024	3 days	Wounds in both horns	Hypermetria, ataxia, head tilt, head pressing, reduced left menace response	Penicillin (40,000 UI/kg), dexamethasone (0.5 mg/kg), thiamine (10 mg/kg)

^a^ Time from clinical signs observed by the owner or handler until physical examination at the hospital. NAD: no available data.

**Table 2:** Laboratory data from 7 ruminants (4 cattle, 2, sheep and 1 goat) with pituitary abscess syndrome.

Parameter/Case	1	2	5	6	7	8	9	RV (Cattle) *	RV (Sheep) *	RV (Goats) *
Hematocrit (%)	33	33	39	29	24	39	33	24–46	24–50	19–38
RBC (×10^6^/µL)	8.1	8.2	10.4	6.1	7.1	8.7	17.4	5–10	8–16	8–18
Hemoglobin (g/dL)	10.4	10.3	12.9	9.4	7.9	13.2	11.3	8–15	8–16	8–14
Leucocytes (/µL)	7300	23,700	9800	10,400	5250	4700	16,850	4000–12,000	4000–12,000	4000–13,000
Segmented neutrophils (/µL)	3212	15,705	4018	3120	4253	1128	15,165	600–4000	700–6000	1200–7200
Bands (/µL)	4065	-	-	104	-	-	-	0–100	0–100	0–100
Lymphocytes (/µL)	1825	6873	4704	3640	893	3337	843	2500–7500	2000–9000	2000–9000
Monocytes (/µL)	438	1185	98	624	105	141	843	25–840	0–750	0–650
Eosinophils (/µL)	-	237	980	2912	-	94	-	0–2400	0–1000	50–650
Fibrinogen (mg/dL)	1000	900	700	600	600	1000	600	200–600	100–500	100–400
STP (g/dL)	7.8	9.4	7	8	6.9	8.6	8.2	6.7–7.4	6–7.9	6.4–7
Albumin (g/dL)	ND	ND	2.1	1.7	0.9	ND	2.1	3–3.5	2.4–3	2.7–3.9
Globulin (g/dL)	ND	ND	4.9	6.3	6	ND	6.1	3–3.4	3.5–5.7	2.7–4.1
Urea (mg/dL)	ND	ND	52	44	57	31	30	42.8–64.2	17.1–42.8	21.4–42.8
Creatinine (mg/dL)	ND	ND	1.4	2.4	1.3	1.3	1.3	1–2	1.2–1.9	1.2–1.8
AST (UI/L)	ND	ND	94	ND	162	609	424	20–34	68–90	43–132
GGT (UI/L)	ND	ND	91	ND	84	23	45	6.1–17.4	20–52	20–56

RBC: red blood cells; STP: serum total protein; AST: aspartate amino-transferase; GGT: γ-glutamyl transferase; ND: not determined; RV: reference values. * Meyer & Harvey [24], Kaneko et al. [25].

**Table 3:** Results of cerebrospinal fluid analysis of 4 ruminants (3 cattle and 1 goat) with pituitary abscess syndrome, including changes in serial samples from one cattle (Case 8) and one goat (Case 9).

Case	Day	Aspect	Color	Density	pH	Proteins (mg/dL)	Pandy	Red Blood Cells (/µL)	Leucocytes (/µL)	Pleocytosis	Bacterial Culture
1	1	Cloudy	Colorless	1.014	ND	500	ND	72	50	Neutrophilic	*Trueperella pyogenes*
2	1	Clear	Colorless	1.008	ND	412	ND	11	17	Monocytic	ND
8	2	Clear	Colorless	1.002	8	34.6	ND	22	13	Monocytic	No growth
	9	Cloudy	Light yellow	1.010	7	187.8	Positive	71	1871	Neutrophilic	*Corynebacterium* sp.
9	2	Clear	Colorless	1.008	7	36.8	Positive	9	4	Absent	ND
	5	Clear	Colorless	1.008	7	54.6	Positive	8	4	Absent	ND
RV *	-	Clear	Colorless	<1.010		<67	Negative	Rare	<10	Absent	No growth

ND: not determined; RV: reference values. * Scott [20,21] and Stokol et al. [22].

## Data Availability

The original contributions presented in this study are included in the article. Further inquiries can be directed to the corresponding author.
